# Quality assessment parameters for EST-derived SNPs from catfish

**DOI:** 10.1186/1471-2164-9-450

**Published:** 2008-09-30

**Authors:** Shaolin Wang, Zhenxia Sha, Tad S Sonstegard, Hong Liu, Peng Xu, Benjaporn Somridhivej, Eric Peatman, Huseyin Kucuktas, Zhanjiang Liu

**Affiliations:** 1 The Fish Molecular Genetics and Biotechnology Laboratory, Department of Fisheries and Allied Aquacultures and Program of Cell and Molecular Biosciences, Aquatic Genomics Unit, Auburn University, Auburn, AL 36849, USA; 2 Key Laboratory for Sustainable Utilization of Marine Fisheries Resources, Ministry of Agriculture, Yellow Sea Fisheries Research Institute, Chinese Academy of Fishery Sciences, Qingdao 266071, PR China; 3 Bovine Functional Genomics Laboratory, United States Department of Agriculture, Agricultural Research Service, 10300 Baltimore Avenue, Beltsville, Maryland 20705, USA

## Abstract

**Background:**

SNPs are abundant, codominantly inherited, and sequence-tagged markers. They are highly adaptable to large-scale automated genotyping, and therefore, are most suitable for association studies and applicable to comparative genome analysis. However, discovery of SNPs requires genome sequencing efforts through whole genome sequencing or deep sequencing of reduced representation libraries. Such genome resources are not yet available for many species including catfish. A large resource of ESTs is to become available in catfish allowing identification of large number of SNPs, but reliability of EST-derived SNPs are relatively low because of sequencing errors. This project was designed to answer some of the questions relevant to quality assessment of EST-derived SNPs.

**Results:**

wo factors were found to be most significant for validation of EST-derived SNPs: the contig size (number of sequences in the contig) and the minor allele sequence frequency. The larger the contigs were, the greater the validation rate although the validation rate was reasonably high when the contigs contain four or more EST sequences with the minor allele sequence being represented at least twice in the contigs. Sequence quality surrounding the SNP under test is also crucially important. PCR extension appeared to be limited to a very short distance, prohibiting successful genotyping when an intron was present, a surprising finding.

**Conclusion:**

Stringent quality assessment measures should be used when working with EST-derived SNPs. In particular, contigs containing four or more ESTs should be used and the minor allele sequence should be represented at least twice. Genotyping primers should be designed from a single exon, completely avoiding introns. Application of such quality assessment measures, along with large resources of ESTs, should provide effective means for SNP identification in species where genome sequence resources are lacking.

## Background

Most performance traits of agricultural relevance are complex traits that are governed by multiple genes. Due to the large number of genes underlying a single trait and their complex interactions, direct genetic analysis of such traits has been difficult. In the past decade, genetic mapping has demonstrated great promise for the analysis of complex traits. In particular, wide applications of microsatellite markers in animal genome studies have allowed major progress in understanding of genes underlying major performance traits [1,2]. However, as larger genome datasets have become available recently, it is clear that microsatellites are not sufficiently dense to provide the genome coverage necessary for the dissection of many of the highly complex traits such as disease resistance, feed conversion efficiency, growth, and carcass traits. In addition, large-scale automated genotyping of microsatellites has not been possible. Recently, much excitement was generated with the ability to analyze complex traits with new types of polymorphic markers. Efforts have shifted to single nucleotide polymorphisms (SNPs).

SNPs are the most abundant type of genetic variation. Theoretically, SNPs can have four alleles, but they have been regarded as bi-allelic as most often, only two alleles have been observed at a given position [3]. SNPs are estimated to occur once every 500 to 1,000 bp in humans when any two chromosomes are compared [4-6] and their frequencies have been estimated to be higher in other organisms [7,8]. This would make it possible to construct genetic maps with extremely high marker densities allowing identification of haplotypes using SNPs, especially for the species with a draft genome sequence [9]. In addition, SNPs offer several other advantages over other molecular markers. First, SNPs themselves are the fundamental causes of the genetic variation and their mapping would provide potential for the identification of the "causing" SNPs as well as the "associated" SNPs with specific and complex traits [10-13]. Second, many technologies have been developed to genotype SNPs cost-effectively in an automated fashion [14-16]. Third, SNPs are sequence-tagged markers with codominant inheritance, suitable for comparative genome analysis [8,17]; and finally, SNPs are highly stable genetic markers compared to tandem repeat markers where the high mutation rates can confound genetic analysis in populations [18,19].

In most cases, genome-wide SNP discovery has relied on the availability of a draft genome sequence where SNPs can be detected from sequences of the two chromosomes of a diploid organism in the sequence assembly. This approach was initially feasible only for humans and model species. However, as the cost of genome sequencing decreases, now draft genome sequences have become available for several agriculturally important species including bovine, chickens, horses, and soon the swine and tilapia. However, for most aquaculture species, it may take a while for the generation of entire genome draft sequences. Facing the equal challenge, alternative approaches must be sought. Hayes et al. (2007) was able to identify a large number of SNPs from EST resources in Atlantic salmon, and they recently demonstrated mapping of EST-derived SNPs to genetic linkage map [20,21]. Their pioneering work with an aquaculture species set a great model for use of ESTs for the identification of SNPs, especially in non-model species [20-22]. In addition, BAC end sequences (BES) can also serve as sources for the identification of SNPs, and the combination of EST and BES could improve the SNP discovery accuracy comparing using only EST sequences [23].

Catfish is the most important aquaculture species in the United States representing over 60% US aquaculture production. Much progress has been made [24] in its genome resource development including a large number of polymorphic markers [25-27], construction of genetic linkage maps [28,29], construction and characterization of BAC libraries [30,31], and construction of BAC contig-based physical maps [32,33]. Particularly, in relation to SNP discovery, a number of genome resources have been developed including approximately 60,000 BAC end sequences [26,27], over 55,000 ESTs [34] and over 400,000 ESTs are being generated by the Joint Genome Institute of the Department of Energy. Such EST sequences will provide an enormous resource for SNP identification. However, as most researchers have experienced, identification of SNPs using ESTs is not without problems. The most frequent problem is the high rate of sequencing errors that lead to the identification of pseudo-SNPs, leading to subsequently great efforts and expense. The objective of this project was to develop a strategy for rapid and reliable identification and evaluation of qualities of EST-derived SNPs to reduce the rate of pseudo-SNPs resulted from sequence errors typically found in single-pass EST datasets, especially those deposited in NCBI where sequence trace files may or may not be available.

## Methods

### EST clustering and contig assembly

All catfish EST sequences were downloaded from NCBI dbEST database, including those of blue catfish and channel catfish. CAP3 was used to assemble the contigs with the parameters set at "minmatch 50, overlap similarity 0.95", to have a minimal overlap of 50 bases and a minimal similarity of 95% [35]. For each contig generated from the CAP3 assembly, BLASTX was conducted against the non-redundant NR database to assist identification of any related ESTs in different contigs. A significant hit was defined as having an E-value below e^-10 ^and 100 minimum of alignment length for all sequences. Following initial gene identification, related ESTs were further evaluated by manual inspection of the alignments.

### SNP identification using EST resources

The autoSNP program was used to detect putative SNPs from the EST sequences [36]. The program utilized the CAP3 output files as input to detect SNPs based on the base redundancy in the sequence alignments. The autoSNP program generated two text files, a contig file including contig ID, consensus length, number of sequences in the contig, and the number of SNPs, a SNP file including Contig ID, SNP position, minor allele frequency, SNP allele, mutation type, and base alignment in the SNP position. The program also generated an *html *file for each contig, including the alignment information and SNP information. With the autoSNP program, the parameters for minimum minor allele frequency for SNP detection varied with the contig size (the number of sequences in the contig): 1) a sequence variation is declared as a SNP whenever a mismatch is identified within contigs with four or fewer sequences; 2) a sequence variation is declared as a SNP when the minor allele sequence existed at least twice within contigs with 5–6 sequences; 3) a sequence variation is declared as a SNP when the minor allele sequence existed at least three times within contigs with 7–8 sequences; 4) similarly a sequence variation is declared as a SNP when the minor allele sequence existed at least four times within contigs with 9–12 sequences, and 5) when the minor allele sequence existed at least five times within contigs with 13–16 sequences and so on.

### Selection of SNPs for this project

To evaluate the effect of contig size and minor allele sequence frequency on SNP reliability, the SNPs with different contig sizes and minor allele frequencies were selected for SNP validation. After initial submission of a set of SNPs to Illumina, GoldenGate assay functionality and designability scores were given by Illumina. SNPs with a range of functionality and designability scores were chosen for evaluation in this project. A total of 384 SNPs were selected for this project. Hot spots of SNP occurrence that may have been caused by low sequence quality were selected to test how sequence quality affects SNP genotyping and validation rates. In addition to sequence quality, the effect of intron presence on genotyping and validation rates was tested by including SNPs with four known genomic sequences.

### Fish samples used for validation of SNPs

A panel of 192 samples were used for genotyping and validation of SNPs including 66 fish from our interspecific mapping resource family F1-2 × Channel catfish-6 (64 backcross progenies plus their two parents), and 21 fish each from three wild channel catfish populations and three domestic channel catfish populations [37].

### SNP genotyping assay

Genomic DNA (250 ng per sample) was used as template for SNP genotyping using the Illumina's bead array technology according to the manufacturer's protocol for GoldenGate assay [16]. Briefly, two allele-specific primers labeled with Cy3 (P1) or Cy5 (P2) and a third locus-specific primer (P3) with an address sequence were first hybridized to the template and allele-specific primers were extended to cross the SNP site to reach the locus-specific primer. After this allele-specific extension, ligation was conducted between allele-specific primer(s) and the locus-specific primer, creating a PCR template. PCR reaction was conducted using both allele-specific primers and the locus-specific primer. The PCR reaction products were hybridized onto a chip (Illumina Inc., San Diego) containing bead types coated with oligo-nucleotides complementary to the locus-specific primer address on the PCR product. Each bead type is represented with an average redundancy of 30× on the array to optimize the accuracy of the final genotype signal. Following hybridization, the bead array signal was determined using a bead array reader, which could convert images to intensity data. The intensity data for each SNP for each sample was normalized and assigned a cluster position (and resulting genotype), and a quality score for each genotype was generated. Final genotyping results were automatically generated for downstream analysis using the BeadStudio software (Illumina Inc., San Diego).

### Data analysis

The BeadStudio software was used to analyze the SNPs data. The dye intensities are examined by the software to determine the genotype of each sample for that locus. A locus returning predominantly signal from Cy3 is AA, Cy5 is BB and an equal signal of Cy3 and Cy5 represents a heterozygous individual. Data is returned with the allele call for each locus as well as a Gentrain score, a measure that represents the reliability of that genotyping call. GenTrain scores was used to measure the reliability of SNP detection based on the distribution of genotypic classes, and the calling frequency was used to measure the successful SNP calling rate from all samples [15]. For this study, GenTrain score of 0.4, call rate of 90%, and minor allele frequency of 0.05 was used. After removing failed SNPs, the remaining SNPs were identified as successful SNPs in genotyping. Successful genotypes were used further for the analysis of minor allele frequencies, and for the calculation of SNP validation rate. Heterozygosity is defined with the formula H = 1-(p_a_^2^+p_b_^2^) where P_a _is the allele frequency of the major allele and P_b _is the allele frequency of the minor allele [38].

## Results

### Sequence Assembly

A total of 54,960 catfish ESTs available from GenBank including 44,437 ESTs from channel catfish and 10,523 ESTs from blue catfish were subjected to cluster analysis to identify putative SNPs. The contig assembly resulted in 5,670 contigs with an average size of 5.5 sequences per contig and an average length of 1,001 bp per contig. The assembly included 3,003 contigs with 2 ESTs, 980 contigs with 3 ESTs, and 1,687 contigs with 4 or more ESTs (Table 1).

**Table 1:** Summary of the EST Assembly

Number of sequences for assembly	54,960
blue catfish	10,523
channel catfish	44,437
Number of contigs	5,670
Number of singletons	23,598
Number of putative transcripts	29,268
Average contig size	5.5
Average contig length (bp)	1,001
No of contig with:	
2 ESTs	3,003
3 ESTs	980
4 ESTs	468
5 ESTs	263
6–10 ESTs	469
11–20 ESTs	246
21–30 ESTs	95
31–50 ESTs	72
> 50 ESTs	74

### Putative SNP discovery

Among 5,670 contigs, SNPs were detected in 4,387 contigs while 1,283 contigs did not include any SNPs. The vast majority (73%) of the SNPs were identified from contigs with 2–3 sequences, the remaining SNPs were identified from contigs with 4 or more sequences (Table 2). A total 33,594 SNPs were identified from the 4,387 contigs, an average of 0.79 SNPs per 100 base pair. The putative SNP frequencies varied greatly among contigs of different sizes ranging from 0.3 to 1.27 SNPs per 100 base pairs. It was apparent that the putative SNP frequency was greater within contigs containing fewer ESTs, an indication of significant sequence errors in contigs of 2 sequences (0.68 SNP per 100 bp), 3 sequences (1.02 SNPs per 100 bp), and 4 sequences (1.27 SNP per 100 bp). Clearly, this is also related to the parameters used in the AutoSNP software where any sequence variation is defined as a SNP in contigs of 2 sequences (1:1), 3 sequences (1:2), and 4 sequences (1:3 and 2:2) whereas the minor sequence allele must be at least twice with contigs of 5–6 sequences, at least 3 times with 7–8 sequences, etc. This observation, while within expectation, strongly demands validation of SNPs identified from EST sequences, especially with small contigs.

**Table 2:** Initial identification of SNPs as detected by AutoSNP software

**No. of Sequences in each contig**	**No. of contigs with SNPs**	**No. of total SNPs**	**Total Consensus Length (bp)**	**SNP frequency (per 100 bp)**
2	2,488	15,220	2,253,452	0.68
3	928	9,314	914,950	1.02
4	458	6,423	506,023	1.27
5	98	361	104,164	0.35
6–10	168	538	179,846	0.30
11–20	69	246	72,058	0.34
21–30	49	220	56,804	0.39
31–50	58	317	69,615	0.46
> 50	71	955	93,065	1.03
Total	4,387	33,594	4,249,977	0.79*

*Average SNP frequency per 100 bp.

### Validation of SNPs

**Figure 1:**
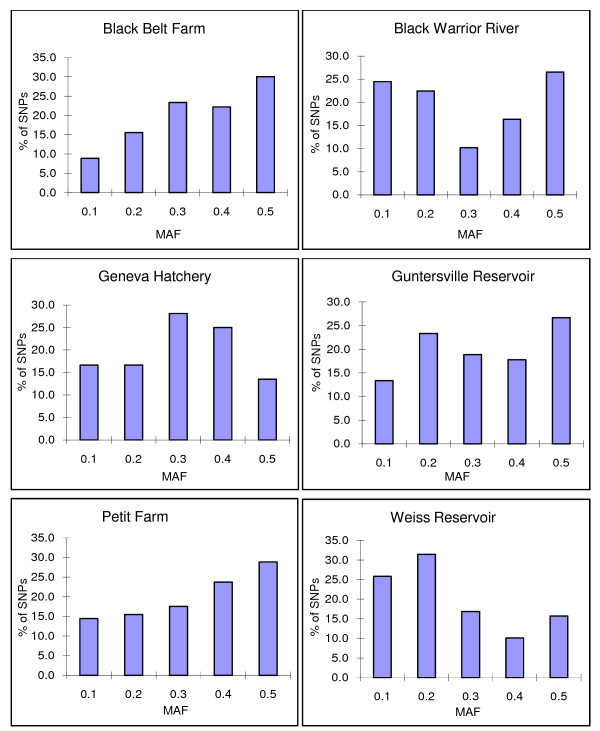
**Distribution of minor allele frequency in domestic and wild channel catfish strains**. The name of the populations is labeled on the top of each panel. MAF: minor allele frequency.

To validate the putative SNPs identified from the ESTs, genotyping using the Illumina Bead Arrays was conducted with 192 fish including 21 fish each from three strains of domestic catfish, and 21 fish each from three wild populations collected from different watersheds, and 66 fish from the inter-specific mapping panel. Of the 266 successful genotyped SNPs, 156 (58.6%) were polymorphic among these 192 fish [see Additional File 1]. Of the 156 SNPs that were polymorphic, 90 were polymorphic in Black Belt domestic population, 96 were polymorphic in Geneva domestic population; 97 were polymorphic in Petit Farm domestic population; 49 were polymorphic in Black Warrior River population; 90 were polymorphic in Guntersville Reservoir population; and 89 were polymorphic in Weiss Reservoir population (Figure 1). Interestingly, the minor allele frequency appeared to be similar in domestic and wild populations.

### The Illumina's Quality Scores of SNPs did not affect SNP validation rates

Of the total of 384 SNPs tested, SNPs were selected with Quality Scores ranging from 0.5 to 1.0. As shown in Table 3, successful genotypes were obtained from 266 SNPs (of which 156 were polymorphic), while genotyping failed for 118 SNPs. Obviously, this failure rate is high, but this did not represent the overall success rate of SNP genotyping using SNPs derived from ESTs as we designed in the experiment several parameters to test SNP quality that obviously lowered the overall success rate. The very obvious question was if the Illumina's Quality Scores (as a reflection of the flanking sequence complexity and sequence context) affected the success rate. As indicated in Table 3, clearly the Quality Scores was not associated with the failures of SNP genotyping as the average Quality Scores for failed SNPs were actually slightly higher than the successful ones.

**Table 3:** Overall summary of the EST-derived SNP genotyping using the Illumina Bead Array technology

**Categories**	**Number of SNPs**	**Average Quality Score**
Successful genotype calling	266	0.87
Polymorphic SNPs	156	0.87
Non-polymorphic SNPs	110	0.87
Failed SNPs	118	0.90
Total number of loci tested	384	0.88

### Contig size and minor sequence allele frequency were the major determinants on SNP validation rates

The percentage of putative SNPs that was validated to be real (SNP validation rate) was found to be directly correlated with contig sizes (number of sequences in the contig) and the minor sequence allele frequencies (Table 4). In general, the smaller the contig size, the lower the SNP validation rate was. However, consistently high SNP validation rate was obtained with contigs of at least 4 sequences with minor sequence being present at least twice. Great differences were observed within contigs with 4 sequences. While SNP polymorphic rate of 70.5% was achieved with contigs of sequences with two sequences of equal frequency (2:2), contigs of 4 sequences with 3:1 sequence frequency had only 15.4% SNP validation rate, suggesting that the minor sequence allele frequency is crucially important. Overall, the average SNP validation rate was only 33.3% for contigs of 4 or fewer sequences with minor sequence allele present only once. However, the overall SNP validation rate for contigs of 4 or more sequences with minor sequence allele present at least twice was 70.9%, and up to 89.2% with contigs of 12 or more sequences (Table 4). Contig length was found not to be related with SNP validation rate. The average contig length of polymorphic SNPs was 1095 bp; the average contig length for monomorphic SNPs was 1071 bp; and the average contig length for failed SNPs was 1080 bp.

**Table 4:** SNP polymorphic rates as a function of contig size and minor sequence allele frequency

# of sequences in the contig	# Successful Loci	Sequence ratio*	Minimal Minor Sequence Frequency	Polymorphic rate (%)
2	24	1:1	50%	33.3
3	37	1:2	33.3%	45.9
4	26	1:3	25%	15.4
Subtotal	87			33.3*
4	44	2:2	50%	70.5
5–6	60	2:3 & 2:4 & 3:3	33.%	60.0
7–8	17	3:4 & 3:5 & 4:4	37.5%	64.7
9–12	21	4:5 & 4:6 & 4:7 & 4:8 & 5:5 & 5:6 & 5:7 & 6:6	33.3%	76.2
>12	37	5:7 & 6:6 & 5:8 & 6:7......... & 12:57	17.4%	89.2
Subtotal	179			70.9*

Total	266			58.6*

*Average polymorphic rate in respective categories.

### Quality of sequences flanking SNPs is important

Flanking sequence quality greatly affected the SNP success rate. Among the contigs with SNPs, we identified 28 contigs with hot spots of SNP occurrence where a region of sequence was highly variable with many "SNPs" detected. Sequence quality examination suggested low quality scores in the sequencing reactions. We intentionally included these SNPs in this project. Of the 28 SNPs tested, 14 (50%) failed in genotyping, suggesting that high sequence quality is required in the SNP region as they are involved in the genotyping primer binding regions (data not shown).

### The presence of intron(s) was the major cause for SNP genotyping failures

The presence of introns greatly reduced the SNP genotyping success rate. Among the contigs containing SNPs, 4 had genomic DNA information that allowed us to test if the involvement of introns has any effect on SNP genotyping and validation rates. All four SNPs failed to provide genotypes. Clearly, The Bead Array technology depends on very short extension and subsequent ligation for success.

Of the 118 failed SNPs, 14 were likely caused by low sequence quality flanking the SNP sites; and 4 were caused by the involvement of introns, as designed in the experiment. The causes for failure of the remaining 99 SNPs were then explored by *in silico *comparative analysis. Based on the fact that intron involvement led to the SNP genotyping failures, we conducted comparative sequence analysis of the catfish ESTs with corresponding zebrafish genes as references. The rationale is that if the gene organization is similar in catfish and zebrafish, then sequence similarity comparison would allow the location of SNP sites to be aligned to the zebrafish genome. If the SNP sites are close to the exon-intron junction, then that could have caused the genotyping failures, assuming conservation of gene structure and organization between catfish and zebrafish. As shown in Table 5, 92 of the 99 catfish SNP loci had significant BLAST hits with the zebrafish genome, but of these, only 50 allowed sequence alignment in the region containing the involved SNPs. Sequence alignment and gene structure in zebrafish indicated that 32 (64%) of the 50 SNPs were located at the exon-intron border, suggesting that the presence of the presumed introns was the major cause for the failures of the SNP genotyping.

**Table 5:** Effect of low sequence quality (as defined by the presence of hot spots of SNP occurrence) and the presence of predicted intron on success rate of SNP genotyping

	**Tested**	**Succeeded**	**Percentage**
Number of loci with SNP located in regions containing low quality sequences	14	7	50%
Number of loci with known introns	5	5	100%
Number of failed loci without gene information	99		
With Significant Blast hits	92		92.9%
SNP positions can be located by similarity comparisons with zebrafish genome	50		54.3%
Number of Loci with SNP predicted to be positioned at exon-intron border	32		64%
Total number of loci potentially with SNP positioned at exon-intron border	37		67.3%

## Discussion

ESTs were proven to be efficient resources for putative SNP identification [20,22,39-41]. This study provides an assessment of nucleotide diversity in available catfish EST resources for putative SNP identification. Since our goal was to make quality assessment for the EST-derived SNPs, we designed this project to provide some answers as to how the sequence context (Illumina's Quality Score), contig size, minor sequence allele frequency, sequence quality flanking SNPs, and the distance between the SNP genotyping primers affect the SNP validation rates.

As compared with SNPs identified from genomic sequences, EST-derived SNPs have several advantages. Since ESTs are transcribed sequences, EST-derived SNPs are associated with actual genes allowing use of gene-associated SNPs for mapping and subsequent use in comparative genome studies [42]. This is particularly important for species without a genome sequence such as aquaculture species. In addition to be used as markers for mapping, SNPs are also considered a rich source of candidate polymorphisms underlying important traits leading to the identification of causative genes or quantitative trait nucleotide (QTN) [43]. However, there are several important factors needed to be considered when using EST-derived SNPs. The major issue for development of SNPs from EST resources is not whether SNPs can readily be identified, but to what degree these SNPs would be reliable, because parameters for quality assessment of EST-derived SNPs simply do not exist. This reliability issue was mostly due to sequence errors; assembled contigs with sequence variation could simply be sequence errors. Additionally, since SNPs derived from ESTs can only be identified from EST contigs where the same gene transcripts were sequenced at least twice and sequencing frequency of ESTs is not random, large scale sequencing is required to identify SNP's from rarely expressed genes. Moreover, SNP rates could be lower in coding regions because of evolutionary restraints of selection pressure.

In this study, over 33,000 putative SNPs were identified from 55,000 catfish ESTs and 384 of these SNPs were tested using 192 catfish samples. We have found that the contig size (number of sequences in the contig) and minor sequence allele frequency were the two major factors affecting the validation rates of EST-derived SNPs. Small contigs had much lower SNP validation rates. Obviously, in small contigs with 2 or 3 sequences, the alternative base is represented only once, and this could be due to sequencing errors. Similarly, in contigs with 4 sequences when the minor sequence allele is represented only once, it is highly likely that the minor allele is due to sequencing errors. We cannot determine the quality of these SNPs without the sequence trace files. Contigs of 4 or more sequences with the minor sequence allele frequency being present at least twice in the contig provided high levels of SNP validation rates (average 70.9% and up to 89.2%). This makes good sense because it is highly unlikely that sequencing errors of two independently sequenced ESTs to occur at the same base location. When at least two ESTs exhibit an alternative base at the putative SNP sites, it is highly likely that such sequence variations are real. All these findings were not unexpected, but for the first time, we provide experimental data to demonstrate the importance of contig size and minor sequence allele frequency. It is noteworthy that even though the larger contigs provided even greater SNP validation rates, contigs of four sequences with even sequence allele distribution (2:2) provided similarly high validation rates. A minimum of two sequences in the contigs representing the minor allele was required to provide a high SNP validation rate [20,22].

**Figure 2:**
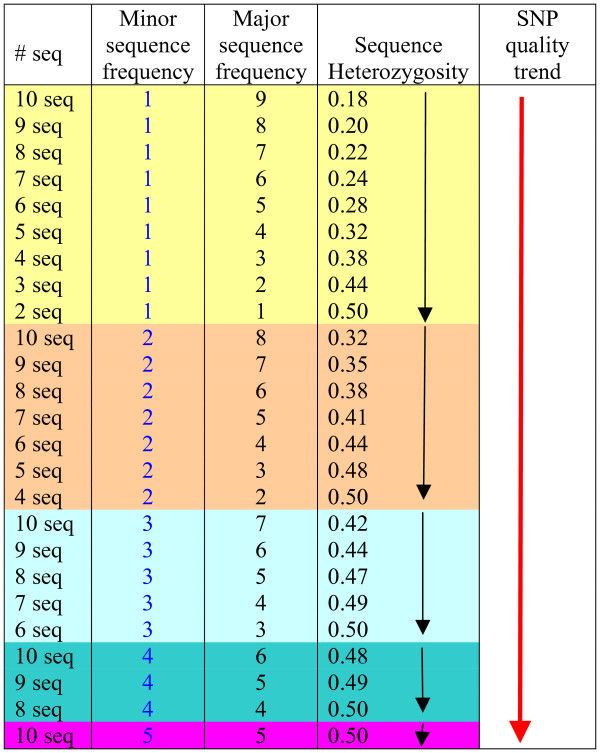
**SNP quality assessment based on EST contig size and sequence frequency of the alleles**. Arrows indicate the trend of SNP quality, with the black arrows indicating trend of heterozygosity within a subset of contigs with the same number of the minor allele sequence, and the red arrow indicating overall SNP quality trend.

The presence of minor allele sequence in relation to the contig size is important. For instance, if the minor allele sequence was present only once, then the smaller the contig size, the more likely the SNP could be real. This is because the contig size of ESTs is simply a reflection of expression abundance. If a rarely expressed gene was sequenced twice, with the alternative allele being present once each, one can still expect that the allele frequency could be equal or close to be equal when the transcript is sequenced 10 times. However, if the transcript was already sequenced 10 times with the minor allele sequence being present only once, it is more likely that the minor allele could have been derived from sequencing errors (Figure 2). This relation is obvious when sequence heterozygosity is considered, as shown in Figure 2. A contig of two sequences with one each of the alternative alleles would have a sequence heterozygosity of 0.5, while a contig with 10 sequences of 9 major allele:1 minor allele would have a sequence heterozygosity of only 0.18.

Another advantage of the SNP identification from EST sequences is its ability to identify the uncommon sequence variants [41]. The monomorphic SNP rate was highly related to the number of samples tested, since the uncommon sequence variants possess very low minor allele frequency, which required a large number of samples. According to our results, the monomorphic SNPs accounted for 28% of tested SNPs. However, these monomorphic SNPs could be false SNPs caused by sequencing errors. The SNPs derived from contigs with four sequences with only one minor allele sequence had the highest monomorphic rate, and the SNPs derived from more than 10 sequences contigs had the lowest monomorphic rate, suggesting that most of the monomorphic SNPs could have also derived from false SNPs, not uncommon sequences variants. In addition, much smaller fish samples (10 fish) were used to construct the EST libraries than the number fish samples used here to validate the SNPs, further supporting the possibility of sequencing errors related to monomorphic SNPs.

Sequence quality flanking the SNP sites was found to be important for successful SNP genotyping using Illumina's Bead Array technology, but not the flanking sequence context as referred to as the Quality Score by Illumina when above 0.5. It is probably true that SNP genotyping primers would have worked properly for the most part even if the sequence context was somewhat simple or A/T-rich, or G/C-rich. However, sequence errors in the SNP region could directly affect the base pairing of the SNP genotyping primers. Low quality sequences could easily generate false SNPs, especially at the beginning or at the end of the sequence. Therefore, sequence quality surrounding the SNP site should be used as one parameter to identify reliable SNPs. However, many EST sequences retrieved from NCBI do not have quality scores or trace files. In such cases, greater caution should be exercised. In particular, hot spot of SNP occurrence should be avoided if possible.

**Figure 3:**
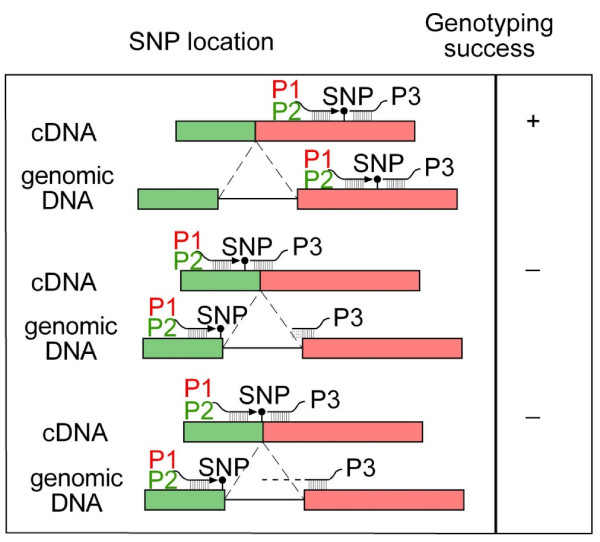
**Schematic illustration of the effect of introns involved in SNP genotyping**. In the first case, all the genotyping primers are located in the same exon nearby, leading to successful genotyping (+); in the second case (middle), one of the genotyping primers (P3 as shown) was located at the exon-intron border, causing non-base pairing that lead to failure of genotyping (-); and in the third case, even though all primers were located in exon regions. However, an intron was involved that demands PCR extension to across the intron. Apparently, the Bead array technology provide very limited extension capability, leading to genotyping failure (-) as well.

Selection of SNPs to allow both allele-specific and locus-specific primers to be located in a single exon is the key to achieving high success rate of SNP genotyping. We found that all tested SNP sites involving introns failed in genotyping. There seemed to be different reasons for such genotyping failures. The most notable cause is that the genotyping primers are located at exon-intron boundary, leading to non-base pairing of the primers with DNA amplified from genomic DNA (Figure 3). In addition, it appeared that the extension of the genotyping primer P1 and/or P2 to reach P3 (see Materials and Methods above) is quite limited. In cases when even both genotyping primers had a perfect match with the template DNA, genotyping failed also simply because an intron was predicted to be present between the genotyping primers (Figure 3). This is somewhat unexpected as one would expect that DNA polymerase should be able to extend easily a few hundred bases. In addition to the few tested loci, comparative gene organization analysis suggested that the vast majority of failed SNPs involved introns immediately flanking the SNP sites, further supporting the inability of genotyping when SNP is located at the exon-intron boundary or when introns are included in the extension reaction. Therefore, bioinformatics analysis using *in silico *comparative sequence and gene structural analysis is important when dealing with EST-derived SNPs.

## Conclusion

In this project, we demonstrated that ESTs are powerful resources for SNP identification. In spite of the development of highly efficient methods for SNP identification from genomic sequences, such as using deep sequencing of reduced representation libraries [44], SNP discovery from EST resources does not require any additional bench work. These existing resources need to be enabled by bioinformatics analysis. EST-derived SNPs are from transcribed genes, and therefore they are applicable to the identification of quantitative trait nucleotide (QTN) as well as to comparative genome analysis [42].

The key to the success of SNP genotyping using EST-derived SNPs is the avoidance of introns. The most important factors for high rate of SNP validation are the contig size (number of sequences in the contig) and the minor sequence allele frequency. Presence of minor sequence allele at least twice in the contig is crucial for EST-derived SNP validation. Use of contigs with at least four sequences, when coupled to the presence of minor sequence allele at least twice in a contig should provide a high level of confidence for the validation of EST-derived SNPs. Quality assessment measures for the EST-derived SNPs presented here should be applicable to EST-derived SNPs from all species. Application of such guidelines, along with the availability of large numbers of ESTs, should make it effective to identify and apply EST-derived SNPs for genome-scale analysis.

## Authors' contributions

WS drafted the manuscript, prepared the samples, and performed the putative SNP bioinformatics analysis and genotyping data analysis. ZS assisted the bioinformatics data analysis and samples preparation. TS conducted all the genotyping experiments. HL, PX, SB, and EP assisted in the putative SNP bioinformatics data analysis. HK assisted in drafting and finalizing the manuscript. ZL served as the P.I. for the overall design and execution of the project, and manuscript preparation. All authors read and approved the final manuscript.

## Supplementary Material

Additional file 1 **Polymorphic SNP markers information**. The file contains the polymorphic SNP markers information including IDs of SNPs, contigs sequences w/SNP sequence and position, and BLASTX hits. Click here for file
